# Ex-Vivo and In-Vivo Assessment of *Cyclamen europaeum* Extract After Nasal Administration

**DOI:** 10.3390/pharmaceutics11090426

**Published:** 2019-08-21

**Authors:** Francisco Fernández-Campos, Beatriz Clares, María J Rodríguez-Lagunas, Olga Jauregui, Isidre Casals, Ana C Calpena

**Affiliations:** 1 Biopharmaceutical and Pharmacokinetics Unit, Pharmacy and Pharmaceutical Technology Department, Faculty of Pharmacy and Food Science, University of Barcelona, Joan XXIII Avenue s/n, 08028 Barcelona, Spain; 2 Pharmacy and Pharmaceutical Technology Department, Faculty of Pharmacy, Campus of Cartuja Street s/n, University of Granada, 18071 Granada, Spain; 3 Physiology Section, Department of Biochemistry and Physiology, Faculty of Pharmacy and Food Science, University of Barcelona, Joan XXIII Avenue s/n, 08028 Barcelona, Spain; 4 Scientific Technological Centers, University of Barcelona, Baldiri i Reixac 10-12, 08028 Barcelona, Spain

**Keywords:** *Cyclamen europaeum*, nasal dosage form, nasal drug delivery, permeability, rhinosinusitis

## Abstract

Rhinosinusitis is a prevalent disorder with a severe impact on the health-related quality of life. Saponins of *Cyclamen europaeum* exert a clinically proven curative effect on rhinosinusitis symptoms when instilled into the nasal cavity, however, more extensive preclinical assessment is required to better characterize the efficacy of this botanical extract. This work evaluates the potential use of a natural freeze-dried extract of *C. europaeum* given as topical nasal administration. Permeation experiment on porcine nasal mucosa was performed with Franz diffusion cells. Experiments in rabbits were performed to test for any toxicological, hematological, biochemical or histological evidence of systemic action. No theoretical levels of saponins were found in the receptor chamber of Franz diffusion cells. Hematological data did not show significant differences between control and experimental animals (*p* > 0.05). Histological studies also showed that enhanced secretory activity in response to intranasal administration was not accompanied by any visible signs of injury. An examination of the brain, lungs, liver, kidneys, spleen, and gastrointestinal organs did not reveal any abnormality. The absence of mucosal permeation of saponins and negligible probability of *C. europaeum* saponins absorption in the course of a therapeutic application was demonstrated.

## 1. Introduction

In recent years, rhinosinusitis (RS) has become a major health problem, and its incidence is steadily increasing around the world with associated health-system costs. RS is an inflammatory disease affecting the paranasal sinus, and it is classified according to duration: acute RS, short-term up to 4 weeks (viral etiology is the most frequent) [1], and long-term which lasts for about 12 weeks or more [2]. It is thought that 75% of patients with acute RS take antibiotics unnecessarily [3]. Clinical symptoms do not always indicate the necessity of antibiotics, and more reliable tools for an efficient RS diagnosis are still needed [4]. As part of the treatment, oral steroids, antihistamines, nasal irrigation, mucolytics, analgesics and local or systemic adrenergic decongestants are usually administered to suppress vascular circulation and gland secretion [5]. The subsequent lack of drainage could worsen the sensation of pressure and microbe accumulation on the sinus. The literature shows little evidence of the effectiveness of such treatments and consequently, alternative therapies are required. In this context, the freeze-dried natural fluid extract of the *Cyclamen europaeum* plant delivered intranasally is thought to have beneficial effects in relieving congestion by facilitating nasal drainage, and it has an anti-inflammatory effect. *C. europaeum* treatment significantly reduced sinus opacification in patients with acute rhinosinusitis (ARS) [6]. The main active ingredients of *Cyclamen* are triterpenoid saponins [7]. A high percentage of curative outcomes has been shown in several clinical trials using *C. europaeum*, demonstrating its effectiveness in the symptomatic treatment of ARS [8].

The main biologically active compounds of *Cyclamen* are triterpene glycosides or saponins, characterized by prominent surfactant properties [9]. The initial action of saponins is confined to a limited area of the vestibule and anterior portions of the inferior concha of nostrils, where the nociceptive endings of ethmoidal nerve are stimulated (a branch of the trigeminus nerve). As a result, secretory and some other reflex responses are elicited. The reflex character of the response is also evidenced by its elimination with local anesthetic spray of 1% dicaine preceding instillation of saponins [10,11,12]. A physical action mechanism has also been described for saponins, since there are surface-active compounds acting as detergents, reducing surface tension on the cell membranes [13]. This effect is the basis of the described hemolytic effect of saponins and could also be responsible for triggering the flushing effect which releases accumulated mucus in the sinuses when it is used to treat RS symptoms.

Several clinical trials have been carried out to assess herbal extract efficacy in RS patients. *Cyclamen* saponins freeze-dried extract reduces the symptoms of acute RS, restores muco-ciliary movements, and improves the rate of remission when compared to a placebo [4]. In these studies, the number of adverse effects was higher in the treatment group, mainly due to irritation at the treatment site. No serious side effects were reported, however, mild events such as nasal and throat irritation, mild epistaxis, and sneezing occurred in the 50% of participants involved in clinical trials compared with placebo treatment, where 24% reported mild events. Other clinical trials have been carried out to assess *Cyclamen* saponins in acute RS combined with antibiotics and in chronic RS with favorable results compared to monotherapy or combination therapies [14,15,16].

Regarding the pharmacokinetic properties of saponins, it is well known that most of them are absorbed so poorly that they produce only local effects; however, there is some absorption (e.g., githagin of *Agrostemma githago*). The most studied saponins in the literature are those derived from *Panax ginseng* and *Glycyrrhiza glabra*, given their well-known therapeutic benefits [17,18]. As generally, they have very low systemic availability, thus a high herbal extract dose is usually required to reach significant blood levels and to achieve a therapeutic effect.

Few cytotoxic studies have been performed on *Cyclamen* spp. [19,20,21]. Moreover, no in-vivo studies were found in the literature describing the toxicological profile of *Cyclamen* saponins after nasal administration.

Considering the dose and structure-related effect on cell viability of saponins in in-vitro studies and the mild adverse effects detected in clinical trials, a more extensive preclinical characterization is required to assess *Cyclamen* saponins. This report describes an ex-vivo experiment in nasal porcine mucosa to determine *Cyclamen* saponins permeability. Moreover, an in-vivo study at an equivalent therapeutic dose and a three-fold dose was conducted in rabbits to check the macro and microscopic effects of *Cyclamen* saponins treatment. No bibliographic references were found describing the mentioned issues. Information on nasal permeability and microscopic evaluation is needed to evaluate the observed adverse effects in humans seen in clinical practice, since *Cyclamen* plant extract is a common treatment in many regions of the world.

## 2. Materials and Methods

### 2.1. Materials

Lyophilized powder of aqueous extract obtained from *C. europaeum* tubers (Nasodren^®^) was kindly supplied by Hartington Pharmaceutical (Barcelona, Spain). HPLC-grade acetonitrile (ACN) and formic acid were purchased from Fluka (Barcelona, Spain). Water was purified by a Mili-Q system (Millipore Iberica S.A.U., Madrid, Spain) until resistivity of 18 MΩ cm was achieved.

### 2.2. Drug Analytical Determination

Each vial of lyophilized powder of *C. europaeum* tubers contained 50 mg of lyophilized extract, which was reconstituted in 5 mL of aqueous solution (water for injection) at a final concentration of 10 mg/mL. Liquid chromatography tandem mass spectrometry (LC-MS/MS) was used as the qualitative determination of saponins in nasal permeation experiments.

Lyophilized powder from *Cyclamen* extract was used as a reference standard since no other standardized extract was available on the market. One vial of the product (50 mg) was dissolved in 5.0 mL of Milli-Q water, and further diluted from 1 to 0.001 mg/mL. Only saponin G was analyzed since it is the main compound found in *Cyclamen* extract.

UPLC Acquity (Waters Corp., Milford, MA, USA) was coupled to an API3000 (AB Sciex, Concord, ON, Canada) mass spectrometer. A mobile phase composed of 0.1% formic acid in water (A) and 0.1% formic acid in ACN (B) passed through a column Symmetry C18 5 µm 3.9 × 150 mm (Waters Corp., Milford, MA, USA) at a flow rate of 0.7 mL/min. The injection volume was 10 µL and the elution was at room temperature according to the following gradient pattern: (t(min), %B): (0, 20), (1, 30), (15, 53), (15.2, 100), (17, 100) with re-equilibration for 5 min.

The saponin responses were measured in the positive ion mode using a TurboIonSpray^®^ source. The mass spectrometer settings were: Nebulizer gas (N_2_) at 10 (arbitrary units); curtain gas (N_2_) at 12 (arbitrary units); auxiliary gas (N_2_) at 6000 cc/min heated to 400 °C; and CAD gas (N_2_) at 4 (arbitrary units). TurboIonSpray^®^ voltage +4500 V; declustering potential (DP) +50 V; focusing potential (FP) +200 V; entrance potential (EP) +10 V; collision energy (CE) 40; and cell exit potential (CXP) 15.

Reconstituted freeze-dried extract viscosity and rheological behavior were determined by means of a RheoStress 1 rheometer (ThermoFisher Scientific, Barcelona, Spain) equipped with a plate-plate system. Measurement was performed at 25 °C.

### 2.3. Nasal Mucosa Permeation Experiment

Three to 4-month-old male and female pigs were purchased from the Animal Facility at the Bellvitge Campus of Barcelona University (Barcelona, Spain). Immediately after the animals (*n* = 6) were sacrificed using an overdose of sodium thiopental anesthesia, the nasal mucosa was surgically removed. The protocol of the study was approved by the Ethical Committee of Barcelona University.

Fresh porcine nasal mucosa from the respiratory region was dermatomed in sections of 400 ± 50 µm thickness (Aesculap GA 630, Tuttlingen, Germany). This thickness is sufficient to represent the entire mucosal barrier of the nasal cavities, including the epithelial barrier (thickness around 100 µm) as well as part of the underlying connective tissue [22]. Membranes were mounted on Franz diffusion cells (Vidra Foc, Barcelona, Spain) with a diffusion area of 0.63 cm^2^. The receptor compartment (6 mL) was filled with Hanks solution, to preserve tissue viability, under magnetic stirring (600 rpm) at 37 °C. The dose placed in the donor compartment (8 µg/cm^2^) was prepared by reconstituting a commercial vial (at a concentration of 0.1 mg/mL water for injection). The sieved drug solution (50 µL) covered the entire mucosal surface of Franz cells. The selected dose was equivalent to the prescribed human dose: one spray (0.13 mL) of 10 mg/mL dilution (50 mg of extract/vial + 5 mL water for injection) covering an effective nasal area of 160 cm^2^ [23].

Samples were removed from the receiver compartment at periods of up to 25 min (1, 2, 5, 7, 9, 12, 15, 20, 25), and the volume in the receiver compartment was replaced by an equivalent volume of fresh medium.

At the end of the permeation study, the mucosa was removed from the Franz cells, cleaned with gauze soaked in a 0.5% solution of sodium dodecyl sulfate, and washed in distilled water. The permeation areas of the mucosa were cut and weighed. Drug content in the mucosa was extracted with 1 mL of water HPLC-grade for 20 min under sonication in an ultrasound bath.

### 2.4. Nasal Mucosa Integrity

In parallel to the previous experiment, two additional Franz cells were mounted with porcine nasal mucosa to evaluate the mucosa integrity after the permeation assay. Integrity evaluation is important in these experiments because the epithelial membrane is the main barrier to the drug passing through mucosa [24]. Moreover, the effect on the epithelium was evaluated. The same drug dose (8 µg/cm^2^) was applied to one cell whereas the other was sieved with 50 µL of water for injection acting as control. A histological evaluation was also carried out. The tissue was embedded in paraffin, cut at 5 µm and dyed using the standard hematoxylin/eosin (HE) procedure.

### 2.5. In Vivo Assessment Evaluation in Rabbits

#### 2.5.1. Multiple Dose Schemes

Chinchilla rabbits (outbred) of both sexes, weighing between 1.5 and 2 kg, were used. Rabbits were housed in the animal facility of the Central Research Laboratory of the Tbilisi State Medical University (Tbilisi, Georgia) in controlled conditions. The study was approved by the ethical committee of the Medical University of Tbilisi. On the day of the experiment, the animals were placed in special boxes with a clamp to immobilize the head. Prior to the extract administration, mucosa sensitivity was determined by administering two drops of a 0.9% NaCl solution (physiological saline solution) 30 min before administration of the study solutions.

Three groups of animals were used (five animals in each); A group rabbits were instilled with 0.13 mL (approximately two drops) of diluted lyophilized powder from the *Cyclamen* extract in water for injection, 10 mg/mL (equivalent to a therapeutic dose) in both nostrils once daily for 4 weeks. B group, was used as an overdose group, and was exposed to a three-fold (by volume) therapeutic dose. C group was used as control, and animals were administered approximately six drops of water for injection.

#### 2.5.2. Animal Evaluation

The treatment effect was studied recording the time of appearance of the secretion in the nostrils and the duration of the reaction. Animals were observed 4 h after administration, and prior to the following administration (24 h) in order to determine any possible sequelae. 24 h after discontinuing treatment, the animals underwent a final general examination.

Before starting the experiment, blood samples were obtained from auricular veins for subsequent hematological and biochemical analyses. Furthermore, 24 h after the completion of treatment, blood was collected by means of heart puncture under urethane anesthesia (2.0 g subcutaneously). The animals were then autopsied and the parenchymatous organs, segments of the digestive and of upper respiratory tracts (including nasal mucosa) and olfactory region of nasal mucosa were fixed, dehydrated and embedded in paraffin for histological examination. Then, tissues were cut (5 µm), rehydrated and stained with hematoxylin and eosine or periodic acid-schiff histochemical reaction (PAS) [25].

The following parameters were studied: (a) overall appearance, survival, food consumption, behavior, and other phenomena observed by means of a general examination; (b) hematology parameters: hemoglobin, red blood cells (RBC) counts, erythrocyte sedimentation rate (ESR), white blood cell (WBC) counts, and platelet counts; (c) blood sample biochemistry: AST/SGOT, ALT/SGPT hh (serum glutamic oxaloacetic transaminase and serum glutamic pyruvic transaminase respectively), total protein, serum bilirubin (total and conjugated), urea, creatinine, glucose, potassium, and sodium.

The hemoglobin was determined by the cyanohemoglobin method, using the Hemoglobin-Agat reagent kit (Agat, Moscow, Russia). Red and white blood cell counts were performed in the Goryaev chamber (EA7. 211.401–Mod. 851, Moscow, Russia). Red cell morphology, platelet and WBC differential counts were performed on blood smears stained with Giemsa-Romanovsky (Med-Service, Kaluga, Russia). Transaminases, urea, creatinine and glucose were measured using the Lachema reagent kit (Erba Lachema, Brno, Czech Republic); serum bilirubin was determined by Jendrassik and Cleghorn’s method [26], total protein with the IRF 454/RL-1 3480 refractometer (PZO, Praga, Poland); sodium and potassium by PFM U 4.2 flame photometer (Russian design), and hemolysis rate by UV SF-26 spectrophotometer (LOMO, Saint Petersburg, Russia).

## 3. Results and Discussion

### 3.1. Analytical Method Development

Injection of samples in both positive and negative ionization modes was done in order to choose maximum sensitivity. The positive mode was chosen to avoid the formation of adducts and clusters which were observed in the negative mode and could have hampered identification.

It was decided to operate in the MRM (multiple reaction monitoring) mode, which is the most suitable mode for quantitative assays using a triple quadrupole mass spectrometer. The SIM (Single Ion Monitoring) mode uses only one quadrupole as m/z filter, and consequently has less specificity than the MRM mode.

**Figure 1:**
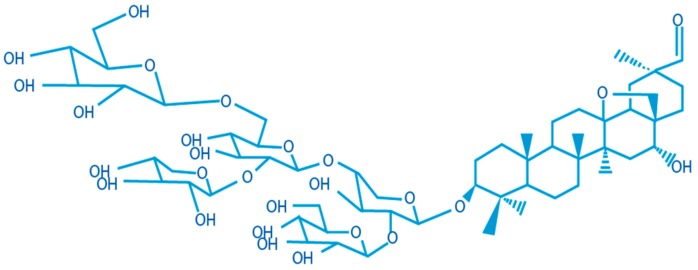
*Cyclamen* saponin G structure.

In the MRM experiments, a whole series of transitions (precursor ion/fragment) are monitored by LC/MS with a dwell time of 75 ms. For quantification purposes, only the signals corresponding to the transitions 1078.3/295.0 and 1239.2/295.2, which are the strongest, have been integrated. These correspond to saponin G (Figure 1) and appear at retention times of 3.7 and 7.1 min.

**Figure 2:**
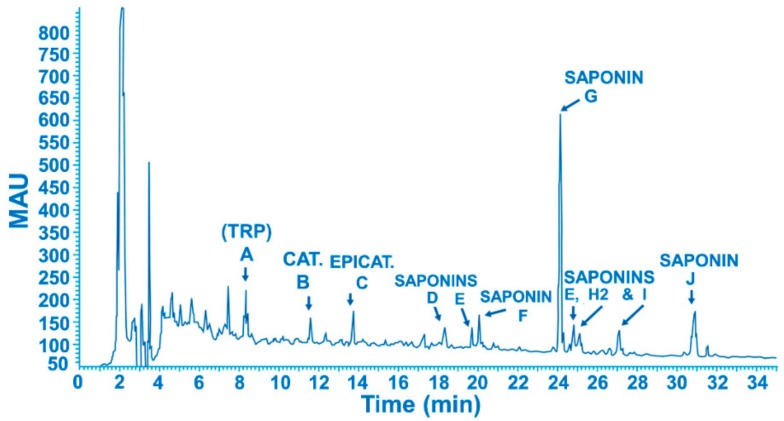
Chromatographic profile of *Cyclamen* freeze-dried formulation.

Saponine G was chosen as a tracer for quantification purposes because it is the main constituent of *Cyclamen europeaum* extract. Chromatographic profile of the freeze-dried extract is shown in Figure 2, in which, it several peaks can be observed. Peak A, is tryptophan. The HPLC analysis of isolated peak A matches that of the tryptophan reference standard. Peaks B and C have identical UV spectra, which are consistent with catechin and epi-catechin (MW 290). The HPLC analysis of isolated peaks B and C matched those of catechin and epicatechin reference standards. Peak D was identified as a saponin of MW 1205 (C_58_H_92_O_26_). Peak E was identified as a saponin of MW 428 (C_28_H_41_O_3_). Peak F was identified as a saponin of MW 1239 (C_58_H_94_O_28_). Peak G was identified as a saponin of MW 1220 (C_58_H_92_O_27_). Peak H was identified as a saponin MW 1221 (C_58_H_92_O_27_). Peak I was identified as a saponin. The latter is a mixture of two structures, saponin I1 of MW 1058.53 (C_52_H_82_O_22_) and saponin I2 of MW 1222.60 (C_58_H_94_O_27_).

The reconstituted extract with 5 mL of water for injection has a pH value of 5.6 and a viscosity value of 0.854 ± 0.05 mPa·s. The rheological behavior is a Newtonian fluid (Figure S1).

**Figure 3:**
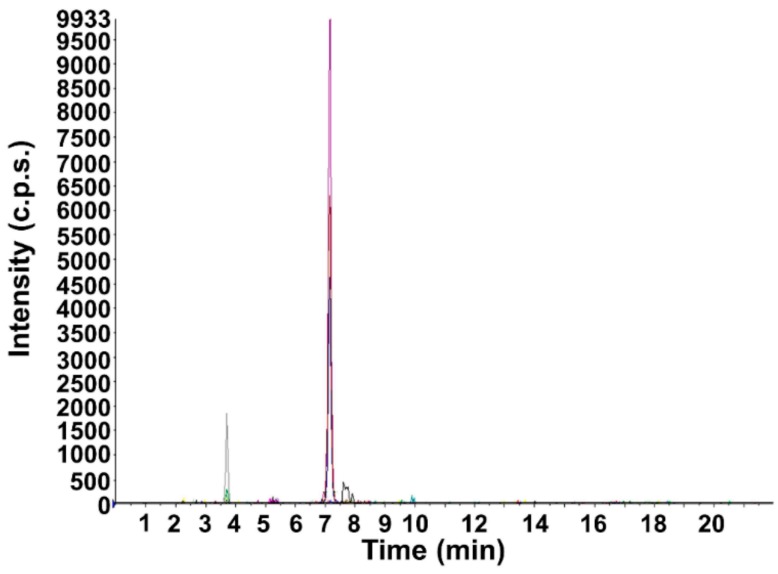
MRM chromatogram of *Cyclamen* freeze-dried formulation.

Moreover, Figure 3 shows a *Cyclamen* lyophilized extract diluted × 2000 (MRM mode).

### 3.2. Nasal Mucosa Permeation Experiment

Ex-vivo mucosa permeation studies were performed to determine whether *Cyclamen* saponins were able to pass through nasal mucosa (specifically the respiratory region). The retained amounts of saponins in the mucosa tissue were also determined. LC-MS/MS analysis showed that no saponins concentrations were observed in the receptor compartment, nor in drug samples retained in the mucosa. There was only one small signal at retention time 7.07 min in one replicate of the drug retained in the mucosa. *Cyclamen* saponins (Figure 1) exhibit an amphiphilic structure, with a glucoside polar section and a lipophilic section with a triterpene group, and a molecular weight over 1000 Da (Section 3.1). These facts mean that saponins in general have poor membrane permeability in accordance with Lipinski’s five rules [27] and, therefore, low potential bioavailability [18].

The time required to perform the permeation experiment (25 min) was selected based on the time taken for mucociliary clearance (MCC). On average, the mucus was expelled after 9 min. This means that in normal conditions, a physiological process removes any foreign particle or substance within this time [28]. In chronic sinusitis, an increase in nasal clearance time is observed and might be due to mucus accumulation. Moreover, the inflammatory response caused by the pathology produces a more alkaline nasal secretion leading to an increase in ciliary movements [29]. In addition to this, in normal and pathological situations, the nasal epithelium is covered by a mucus layer that protects the underlying tissue and limits drug transmission across it. In this experiment, the mucosa was cleaned and dermatomed after animal sacrifice, and this mucus layer was then removed. This situation represented a worst-case scenario: a static model with high contact time of the drug with the mucosa and the absence of a mucus protective layer. Since no drug traces were found in the receptor fluid nor accumulated on the tissue, the likelihood of systemic absorption is low. It is important to bear in mind that a significant amount of the nasal administered dose is swallowed, and gastrointestinal absorption could take place. This issue was not assessed in this study, but considering the usually low prescribed dose of 2.6 mg (one pulverization in each nasal cavity, 0.13 mL/pulverization, of reconstituted extract 10 mg/mL) and the low bioavailability of saponins (between 0.1–1%) [18], the probability of systemic exposure is low. Taking into consideration the described mechanism of action of *Cyclamen* saponins and the flushing effect described in Section 3.3.1, part of the applied dose will be eliminated together with the induced secretion, reducing possible systemic drug exposure even more. In addition, the expected residence time of the administrated extract in the nasal cavity is low, considering the low viscosity of the reconstituted formulation (0.854 mPa·s) and the lack of mucoadhesive polymers in the formulation. Therefore, the maximum presence of the product would be equivalent to the MCC.

**Figure 4:**
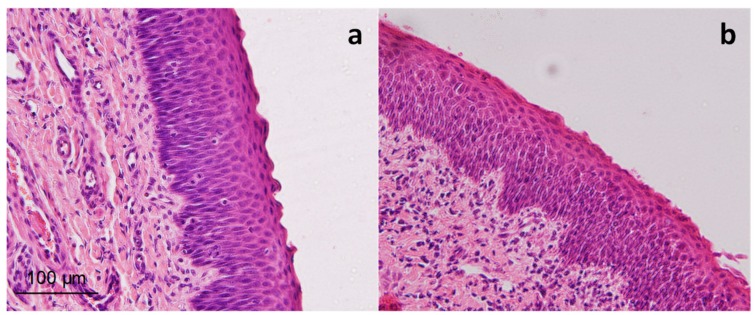
Epithelium structure of nasal porcine mucosa after ex-vivo permeation test. Control mucosa (**a**) and mucosa treated with *Cyclamen* freeze-dried solution at a human-equivalent dose (**b**) stained with hematoxylin and eosin at 200× magnification. Scale bar = 100 µm.

The mucosa tissue was kept in Hank’s solution, a nutritive solution which preserves epithelium viability, one of the critical points in ex-vivo permeation experiments. Previous studies have demonstrated the ability of this medium to maintain buccal mucosa viability [30]. Figure 4 shows the histology of the porcine nasal mucosa treated with the drug formulation after the end of the permeation experiment. There are no signs of epithelial vacuolization, and intercellular spaces are not widened, so treatment does not alter the epithelium morphology. No differences were observed between experimental and control samples.

### 3.3. Multiple Dose Schemes

Multiple-dose administration was performed to simulate the situation in clinical practice. The dose administered corresponds to 0.13 mL of a dilution of 10 mg/mL of lyophilized powder of the *Cyclamen* extract in water for injection.

#### 3.3.1. Macroscopical Evaluation

During the administration of human-equivalent doses (A group), the serous effusion from the nostrils was clearly visible and appeared after 1 or 2 min of instillation; the visible effusion lasted for approximately 15 min. The latent period and duration of the response remained unchanged on subsequent administrations throughout the 4-week course of the test. Previous administrations did not appear to intensify secretion from the nostrils.

In B group (three-fold increased dose), animals expressed a pain reaction such as movements to release the head, licking the upper lips and nostrils, etc. This fact could be attributed to the action of *Cyclamen* saponins, due to their detergent-related activity. However, blood did not appear in the serous effusion. When animals were examined 1, 4 and 24 h after each administration of the therapeutic and three-fold dose, no residual signs of irritation were observed.

Continued administration did not give rise to behavioral deviations, signs of CNS depression, or to alterations in spontaneous motor activity and vegetative functions, e.g., gastrointestinal motor activity such as diarrhea or flatulent intestine, indicative of saponins systemic toxicity.

When the solvent (control group) was administered in the nasal cavity, some rabbits reacted with a snort. However, no secretions or reflex reactions such as sneezing or licking the nostrils were observed, and no movements in an attempt to release their heads were observed, indicating the absence of a pain reaction.

#### 3.3.2. Hematological and Biochemical Examination

Results from the hematological and biochemical parameters are reported in Table 1. No changes were observed in the treated groups (A and B) when compared with the untreated group (C) along the study.

Regarding the hematological data, attention was paid to the RBC counts and the hemoglobin content, which were found unchanged on the completion of the 4-week administration of saponins to both groups of rabbits. If saponins appear in circulation, they characteristically cause hemolysis due to the precipitation of cholesterol in the erythrocyte membranes [17]. Initial signs of membrane damage are manifested by spherocytosis and the appearance of spiculated red blood cells [31]. Such abnormal forms were not detected in peripheral blood smears in either group of rabbits (A and B) treated with the extract. Also, there was no marked polychromatophilia, which would conventionally indicate elevation of reticulocytes suggestive of chronic hemolysis.

Serum unconjugated bilirubin also remained unchanged; this is an interesting parameter in this study because increased values indicate accelerated red blood cell breakdown.

#### 3.3.3. Histological Examination

When animals were sacrificed, an internal general observation was performed. No alterations were observed in the peritoneal and retroperitoneal area, brain, lungs, stomach, intestines, liver or kidneys.

The spleen was also carefully observed since fragile RBC could release hemoglobin which is retained in the reticuloendothelial cords of the spleen causing hyperplasia in pulp cords. Atrophic follicles in white pulp are also representative of chronic hemolysis. None of these conditions were observed in the studied animals.

**Figure 5:**
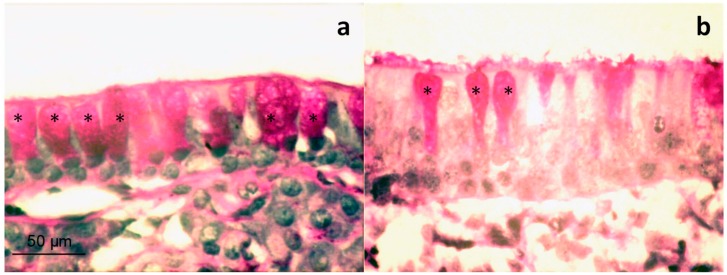
Histology of nasal mucosa (respiratory region) of rabbits treated with freeze-dried *Cyclamen* saponins at therapeutic dose. Different sections shown in panel (**a**) and panel (**b**) are stained with PAS. Asterisks indicate goblet cells full of secretory material. Scale bar = 50 µm.

The examination of respiratory and olfactory membranes has an important interest when nasal products are administrated. In relation to respiratory membranes, intranasal mucus membrane obtained from B group rabbits did not exhibit any change in the structure of the multi-layered (pseudo-stratified) epithelium. The mucus membranes of the tested rabbits showed more intense secretor activity compared with the control animals, particularly when looking at goblet cells, full of mucus, groups of which were detected systematically in the examination (Figure 5a,b). Secretor activity was not accompanied, as a rule, by any signs of inflammation or injury to the epithelial lining of the mucosal surface.

**Figure 6:**
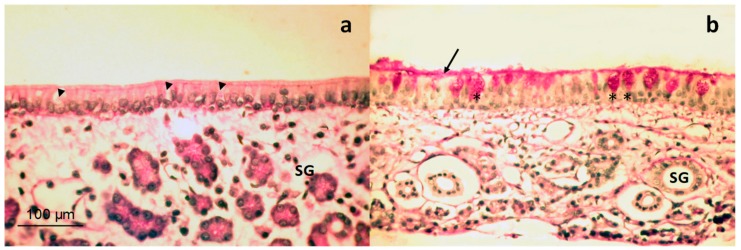
Histology of nasal mucosa (respiratory region) of rabbits treated with three-fold dose. Control mucosa (**a**) without secretory activity and B group mucosa (three-fold dose) administrated animals, (**b**) stained with PAS at 200× magnification. Asterisks indicate goblet cells full of mucus; arrow indicates mucus layer; and arrow heads indicate empty goblet cells. SG = serous gland. Scale bar = 100 µm.

The structure of the seromucous glands is fully preserved in the mucous membrane. The epithelium did not show any alteration; no signs of hyperemia, edema or leukocyte infiltration can be observed (Figure 6b). Control animal mucosa is presented in Figure 6a, with the absence of secretory activity. Thus, secretion caused by the saponins is due to specific action that does not affect the viability and integrity of pseudostratified respiratory epithelium in nasal cavities.

The olfactory epithelium is composed of olfactory sensory neurons, sustentacular cells, and basal cells which are considered as stem cells able to regenerate new sensory neurons. In this regard, the damaged tissue is restored every 1 or 2 months. Therefore, any possible damage to the tissue due to the exposure of the saponins would be restored. In addition, considering that no damage was caused to the nasal mucosa from the respiratory region, no effect would be expected in the olfactory mucosa. Stimuli from the sensory neurons are transmitted through the olfactory nerves to the olfactory bulbs. After the administration of the three-fold dose to rabbits, the histological study of the different neuronal tissues related to the olfactory system showed no alteration of the neurons (Figure S2). Thus, the use of the triterpene *Cyclamen* saponins might be considered safe for olfaction. However, further studies such as behavioral test of olfactory perception should be conducted.

It is also important to note the absence of signs of irritation or inflammatory changes in the mucus membranes of the upper respiratory tract (Figure S3); gastrointestinal tract; and especially of the pharynx, esophagus, larynx, and trachea in animals from B group with pain reaction.

## 4. Conclusions

Triterpene *Cyclamen* saponins were studied ex vivo and in vivo. The ex-vivo nasal permeation experiment showed that no absorption or retention in the tissue occurred with a human-equivalent dose, confirming their low permeability potential and the low likelihood of systemic exposure. In-vivo studies with an administered therapeutic equivalent dose produced a mucosa secretion that lasted approximately 15 min with no signs of itching or irritation. The three-fold overdose produced irritation in rabbits after nasal administration, which is an expected sign considering the purgative effect of the extract, due to the detergent-like structure of *Cyclamen* saponins. No significant macroscopically or microscopical alterations, either hematological or biochemical, were observed. Goblet cells of treated animals appeared full of secretory material, confirming the results of secretory drainage stimulation.

*Cyclamen* lyophilized extract has an adequate safety profile as evidenced by this selected animal model. The adverse effects observed in clinical trials do not seem to be caused by the toxicity of the product and could result from disease-related conditions or increased sensitivity to saponins in some individuals.

## Figures and Tables

**Table 1:** Results from the hematological and biochemical analysis at the completion of the administration of the three-fold dose of *Cyclamen* extract (*n* = 5).

Parameter	Unit	C (Control) Group (Mean ± SD)	B (3-Fold Dose Treated) Group (Mean ± SD)
Red blood cells	10^12^/L	5.90 ± 0.14	5.28 ± 0.40
Hemoglobin	g/L	74.40 ± 14.40	78.90 ± 2.00
Platelets	10^9^/L	319.40 ± 22.30	349.40 ± 38.00
ESR *	mm/h	2.5 ± 0.5	2.5 ± 0.5
Total white blood cells	10^9^/L	8.70 ± 0.23	8.60 ± 0.20
Total bilirubin	mmol/L	3.92 ± 0.90	4.30 ± 0.06
Conjugated bilirubin	mmol/L	0.94 ± 0.40	1.50 ± 0.80
ALT/SGPT **	U/L	66.0 ± 2.22	54.45 ± 4.78
AST/SGOT ***	U/L	74.2 ± 2.79	61.3 ± 3.71
Total protein	g/L	52.96 ± 2.00	58.44 ± 7.40
Urea	mmol/L	3.85 ± 2.77	5.82 ± 3.00
Creatinine	µmol/L	184.40 ± 13.00	167.60 ± 1.60
Na^+^	mmol/L	172.40 ± 3.63	158.60 ± 1.80
K^+^	mmol/L	4.78 ± 0.88	4.81 ± 0.64
Glucose	mmol/L	4.74 ± 1.04	6.67 ± 4.00

* ESR: erythrocyte sedimentation rate; ** ALT/SGPT: Serum glutamic pyruvic transaminase; *** ASP/SGOT: Serum glutamic oxaloacetic transaminase.

## Supplementary Materials

The following are available online at https://www.mdpi.com/1999-4923/11/9/426/s1, Figure S1: Rheological behavior of freeze-dried *Cyclamen* extract reconstituted with 5 mL of water for injection; Figure S2: Images of different neuronal regions implicated in smell from three-fold dose administrated rabbits. Oligodendrocytes of the olfactory tract (a), neurons in the medial olfactory region (b), hippocampal pyramidal neurons (c) and polymorphic neurons of the hippocampal region (d). Hematoxylin and eosin stain, scale bar = 50 µm (a and d) and 50 µm (b and c); Figure S3: Histology of representative epithelial tissues from aerial and digestive tracts from rabbits. Control (a) and three-fold dose treated rabbit (b) Esophagus; three-fold dose treated rabbit larynx specifically epiglottis (c) and trachea (d). Scale bar = 100 µm.
