# Supplementary material for: Ex-Vivo and In-Vivo Assessment of Cyclamen europaeum Extract After Nasal Administration
Source: Pharmaceutics. 2019 Aug 21;11(9):426. doi: 10.3390/pharmaceutics11090426 (PMC6781058; doi:10.3390/pharmaceutics11090426)
Supplement: Supplementary file 1 [file pharmaceutics-11-00426-s001.pdf]

pharmaceutics

Supplementary Materials: Ex-Vivo and In-Vivo Assessment of *Cyclamen europaeum* Extract After Nasal Administration

Francisco Fernández-Campos, Beatriz Clares, María J Rodríguez-Lagunas, Olga Jauregui, Isidre Casals and Ana Calpena

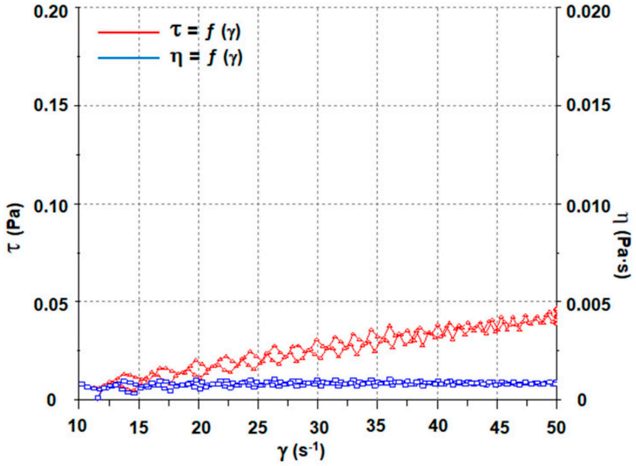

Figure S1. Rheological behavior of freeze-dried *Cyclamen* extract reconstituted with 5 mL of water for injection.

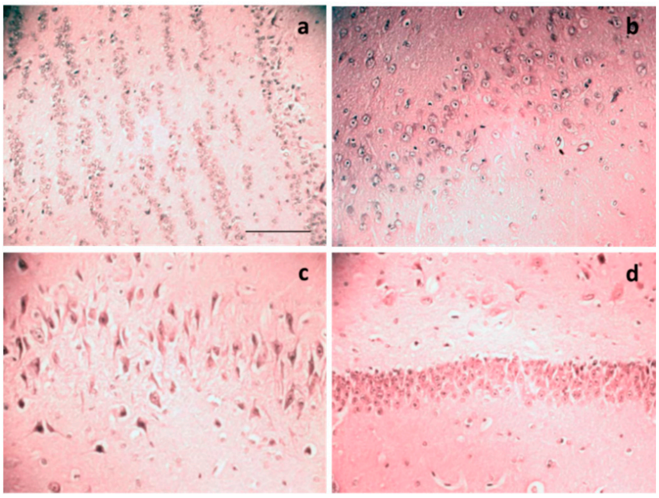

Figure S2. Images of different neuronal regions implicated in smell from three-fold dose administrated rabbits. Oligodendrocytes of the olfactory tract (**a**), neurons in the medial olfactory region (**b**), hippocampal pyramidal neurons (**c**) and polymorphic neurons of the hippocampal region (**d**). Hematoxylin and eosin stain, scale bar = 50 μm (**a** and **d**) and 50 μm (**b** and **c**).

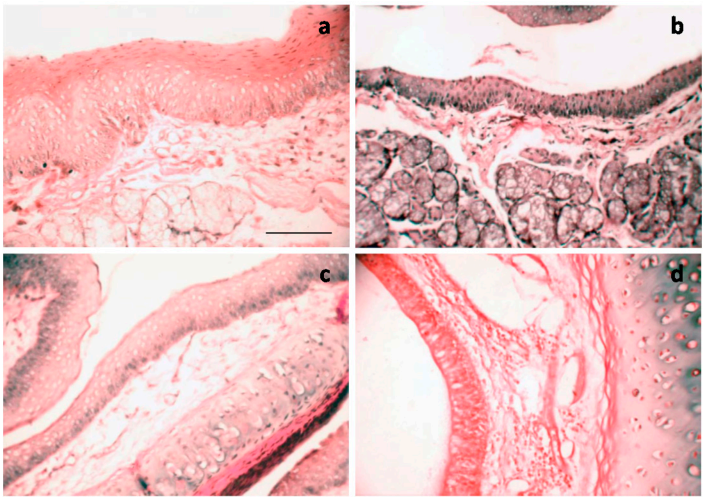

Figure 3. Histology of representative epithelial tissues from aerial and digestive tracts from rabbits. Control (**a**) and three-fold dose treated rabbit (**b**) Esophagus; three-fold dose treated rabbit larynx specifically epiglottis (**c**) and trachea (**d**). Scale bar = 100 μm.
